# Indigenous *Bradyrhizobium* strains enhance nodulation and yield of early maturing soybean in Belgium

**DOI:** 10.3389/fpls.2026.1748102

**Published:** 2026-03-06

**Authors:** Margo Vermeersch, Paul Quataert, Chris Van Waes, Sofie Goormachtig, Niel Verbrigghe, Peter Lootens, Isabel Roldán-Ruiz, Joke Pannecoucque

**Affiliations:** 1 Plant Sciences Unit, Flanders Research Institute for Agriculture, Fisheries and Food (ILVO), Merelbeke-Melle, Belgium; 2 Department of Plant Biotechnology and Bioinformatics, Ghent University, Ghent, Belgium; 3 VIB Center for Plant Systems Biology, Rhizosphere group, Ghent, Belgium

**Keywords:** Glycine max, high-throughput field phenotyping (HTFP), indigenous rhizobia, northwestern Europe, soybean productivity, sustainability

## Abstract

The use of commercial inoculants in Northwestern European soybean cultivation often leads to inconsistent nodulation and yield performance. This study investigates four indigenous *Bradyrhizobium* strains isolated in Belgium, through a combination of controlled growth chamber experiments and multi-year, multi-location field trials. Results show that the indigenous strains can effectively nodulate early maturing soybean varieties (MG00-000) and significantly improve chlorophyll content and key agronomic traits, including shoot biomass, grain yield and protein content. While no significant differences between strains were detected in growth chamber experiments, field trials revealed variation in strain nodulation and yield characteristics, with two indigenous strains, 521_C7_N1.3 and 590_E5_N4.2, performing comparably or even superior to *Bradyrhizobium* strains of commercial inoculants (i.e. G49 and 532C). High-Throughput Field Phenotyping (HTFP) using drones confirms strong correlations between rhizobial inoculation and plant vigor throughout the growing season. This approach provided valuable temporal insights into plant growth dynamics and proved to be an effective complementary tool for assessing inoculant performance. Overall, the findings highlight the potential of indigenous *Bradyrhizobium* strains to enhance soybean productivity in Northwestern Europe, particularly under the climatic and soil conditions of Belgium.

## 1 Introduction

Soybean [*Glycine max* (L.) Merr.] has been an important legume crop in Europe for decades, traditionally valued as a high-protein ingredient in animal feed (Hartman et al., 2011). More recently, interest in soy for human consumption has increased, as its protein provides all essential amino acids and is linked to benefits for both human health and the environment (Grdeń and Jakubczyk, 2023; Messina et al., 2022; Rotundo et al., 2024; van Loon et al., 2023). Despite its importance, limited cultivation forces Europe to rely heavily on imports, raising concerns over food security, sustainability, and environmental impact.

Reducing dependency on overseas soybean imports is a strategic priority under the European Green Deal. Modelling studies suggest that achieving 50% self-sufficiency would be feasible if soybeans were cultivated on 4-5% of the current European cropland (Guilpart et al., 2022). Present production is concentrated in Central and Eastern Europe, with Serbia and Ukraine as the main producing countries. Due to the projected climate change and rising temperatures, soybean cultivation in Central Europe is expected to increase (Nendel et al., 2022). In Northern and Western Europe, however, soybean area remains very limited (FAOSTAT, 2023). Ongoing research therefore focuses on expansion through breeding of locally adapted genotypes, yield and quality improvements and innovations in cropping systems, agronomy and farmer adoption (Farahbakhsh et al., 2025; Fogelberg and Recknagel, 2017; Notz et al., 2023; Pannecoucque et al., 2018b; Watson et al., 2017).

A major advantage of soybeans is their low demand for external nitrogen inputs. Through symbiosis with nitrogen-fixing bacteria, especially *Bradyrhizobium* spp., soybeans can meet 50-60% of their nitrogen needs via biological nitrogen fixation (BNF), with the remainder taken up from soil nitrogen (Salvagiotti et al., 2008). Enhancing BNF is key to improving productivity and promoting sustainable production (Nemecek et al., 2008).

Because of the relatively short history of soybean cultivation in Europe, seed inoculation with rhizobia is essential to ensure adequate nodulation, yield and protein content. Yield and protein content increases due to seed inoculation have been reported across several European regions (Maluk et al., 2023; Pannecoucque et al., 2022). Currently, this is achieved with commercial inoculants that were primarily developed for large soybean-growing regions outside Europe and, where strain identity is available, mainly contain non-European strains. Introducing such strains may lead to suboptimal nodulation and reduced effectiveness under European conditions (Jarecki, 2023; Pannecoucque et al., 2018a; Zimmer et al., 2016).

The inconsistent performance of commercial inoculants with non-native rhizobia in temperate environments (Thilakarathna and Raizada, 2017) has shifted research towards identifying indigenous rhizobial strains, which are hypothesized to be better adapted to local conditions and soybean varieties (Yuan et al., 2020). For example, studies in Africa have shown variable to promising performance of indigenous strains compared to non-indigenous rhizobia under both greenhouse and field conditions (Amani et al., 2020; Chibeba et al., 2017; Desta et al., 2023). In Europe, trials in Germany have demonstrated that inoculating locally adapted soybean varieties with indigenous rhizobia can enhance nodulation, BNF and yield (Omari et al., 2022; Yuan et al., 2020).

Soybean production in temperate regions such as Belgium faces additional challenges. Mean air temperatures during sowing in the month of May range between 9.2 and 18.4 °C based on long-term meteorological records (Royal Meteorological Institute of Belgium, 2025) and suboptimal root-zone temperatures have been identified as key factor contributing to unstable yields and reduced grain protein content (Pannecoucque et al., 2018a). Drought stress further limits nodulation and BNF, with reductions of up to 70% reported (de Freitas et al., 2022; Santachiara et al., 2019). Soil properties such as low pH, salinity and nutrient deficiencies, along with the competitiveness towards the local soil microbiome, also influence symbiotic efficiency (Thilakarathna and Raizada, 2017; Yeremko et al., 2025). In addition to external factors, strong strain × genotype interactions have been documented, highlighting the need to identify effective rhizobia-soybean combinations (Omari et al., 2022; Thilakarathna and Raizada, 2017).

Given these challenges and the lack of regionally adapted inoculants in Western Europe, studying indigenous rhizobia is highly relevant. Identifying strains that combine strong nodulation capacity with resilience to local environmental stresses could help stabilize soybean yield and protein content, while reducing reliance on imported inoculants and external nitrogen inputs.

In this context, the present study evaluated the nodulation efficacy and yield performance of four indigenous *Bradyrhizobium* strains (521_C7_N1.3, 590_E5_N4.2, 1200_B8_N1.2 and 1200_D9_N1.2). Field trials with these strains were previously conducted in 2022, and the effects on nodule number, nodule dry weight, yield and seed protein content were reported earlier (García Méndez et al., 2025). The present study extends this work by including a second year of field trials (2023), assessing additional physiological and agronomic parameters such as chlorophyll content, protein yield and thousand seed weight, and performing dedicated growth chamber experiments. This approach enables a direct comparison between indigenous and self-cultured commercial *Bradyrhizobium* strains G49 and 532C in relation to soil characteristics and weather conditions. Additionally, high-throughput field phenotyping (HTFP) was applied in one of the field trials to assess effects on plant growth, chlorophyll content and plant senescence.

## 2 Materials and methods

### 2.1 Growth chamber experiments

Two consecutive pot trials with an identical setup were executed in a growth chamber under a 16 h light/8 h dark photoperiod and day/night temperatures of 20 °C/10 °C. Relative humidity was maintained between 50-80%. Each experiment lasted eight weeks and was arranged in a randomized block design with four replicates. Six inoculation treatments were tested on five soybean varieties, resulting in 30 pots per block. Pots (4 L) contained a 1:1 (v/v) of sand (particle size 0-2mm; Leus N.V., Melle) and agricultural soil. The soils differed slightly between trials; Trial A: 12.4 mg mineral-N kg^-1^ soil, 10.2 mg P 100 g^-1^ soil and pH-KCl 8.2; Trial B: 8.6 mg mineral-N kg^-1^ soil, 15.6 mg P 100g^-1^ soil and pH-KCl 6.0). Pots were placed on trays to prevent cross-contamination and watered regularly to maintain constant weight. No fertilization was applied during the trial period.

Five early maturing soybean varieties, registered on the European variety catalogue, by different seed companies were selected, i.e. *Glycine max* variety Hermes (MG000, Protealis, Belgium), Acardia (MG000, Saaten-Union, Germany), Lenka (MG00, Prograin, Canada), Aurelina (MG000, Saatzucht Donau, Austria), and Gallec (MG000, Delley Samen und Pflanzen, Switzerland). Seeds were stored at -10 °C until the start of the experiment.

The effects of four indigenous *Bradyrhizobium* strains (521_C7_N1.3, 590_E5_N4.2, 1200_B8_N1.2 and 1200_D9_N1.2), collected within the framework of the *Soy in 1000 gardens* project (García Méndez et al., 2025; Vlaminck et al., 2024), on nodulation and plant vigor were evaluated and compared with *Bradyrhizobium* G49 (Biodoz^®^, De Sangosse, France). Non-inoculated seeds soaked in sterile demineralized water served as control treatment.

All strains were stored at -80 °C in 50% glycerol and cultured on Yeast Mannitol Agar at 28 °C. Liquid cultures were prepared from single colonies to the exponential growth phase (shaken at 175 rpm) and adjusted to OD_600_ = 0.01 with sterile demineralized water. Seeds were immersed in the corresponding suspension for 1 hour before sowing (9 seeds pot^-1^ at ~2.5 cm depth). Watering was standardized throughout the trial to maintain uniform soil moisture.

Two weeks after sowing, seedlings were thinned to three per pot by cutting excess plants, ensuring minimal disturbance to the developing root systems. Eight weeks after sowing, foliar chlorophyll content was measured using a CCM-200 (Opti-Sciences) on three leaves per plant (from basal to apical trifoliate) and the mean of nine readings per pot was used for analysis. Plants were then carefully uprooted, roots rinsed, and nodules counted and assessed for internal color. Shoots and nodules were dried at 70 °C for 72 hours to determine dry weight.

### 2.2 Field trials

#### 2.2.1 Trial description

Five field trials were conducted over two consecutive years (2022-2023) across Flanders, Belgium, to assess the performance of indigenous *Bradyrhizobium* strains on nodulation, soybean yield and protein content. Results concerning nodule number, nodule dry weight, yield, and seed protein content from the 2022 trials have been published previously (García Méndez et al., 2025) and are included here to allow comparison with the newly measured parameters (chlorophyll content, protein yield and thousand-kernel weight) and the 2023 data. Field characteristics and management details are provided in **Table 1**. None of the five fields had a soybean history and no irrigation was applied. Trials followed a randomized complete block design with three replicates. Each plot (10 m × 1.25 m; 12.5 m² gross area) comprised five rows spaced 0.25 m apart and was sown at 65 seeds m^-2^ and 3.5 cm depth. Nets were used for wildlife protection until V1-V2 stages (Fehr and Caviness, 1977). The effective net plot area (8.75 m²) was used for nondestructive and drone-based measurements. Weather data were obtained from the nearest Royal Meteorological Institute stations.

**Table 1:** Soil characteristics and management details of the five field trials conducted in 2022 (Merelbeke and Bottelare) and 2023 (Melle, Merelbeke and Poperinge) across Flanders, Belgium.

Variable	2022	2022	2023	2023	2023
	Merelbeke	Bottelare	Melle	Merelbeke	Poperinge
Coordinates	50.97481, 3.78274	50.97022, 3.75582	50.992556, 3.785667	50.975222, 3.778750	50.898722, 2.787778
Soil texture	Sandy loam	Sandy loam	Sandy loam	Sandy loam	Sandy loam
Precipitation between January 1^st^ and sowing (mm)	149.2	139.5	337.3	337.3	285.4
Precipitation during growing season (mm)	302.7	242.1	301.2	296.9	270.5
Tillage	2022-02-05	2022-03-05	2023-21-05	2023-21-05	2023-30-05
Sowing date	2022-05-05	2022-05-05	2023-25-05	2023-25-05	2023-01-06
Date of chlorophyll content measurement	2022-18-08	2022-26-08	2023-30-08	2023-31-08	2023-28-08
Harvest date	2022-05-10	2022-23-09	2023-11-10	2023-10-10	2023-17-10
Previous crop	Flax	Flax	Summer barley	Potatoes	Potatoes
Assessed varieties	Lenka, RGT Shouna	Lenka, RGT Shouna	Lenka, Acardia, Hermes	Lenka, Acardia, Hermes	Lenka, Acardia, Hermes
Seed inoculation method	Liquid coating	Liquid coating	Vermiculite coating	Vermiculite coating	Vermiculite coating
Soil nutrient status before sowing					
Organic Carbon Content (% dry soil^-1^)	1.16	1.12	1.17	1.31	1.58
Soil pH-KCl	5.8	6.0	5.5	6.1	6.5
Soil mineral nitrogen (N_min_) (0-30 cm) (kg ha^-1^)	13.3	13.4	11.4	8.2	17.4
Soil mineral nitrogen (N_min_) (0-90 cm) (kg ha^-1^)	21.7	33.29	52.2	50.4	74.9
Fertilization					
K_2_O 40% (200 kg ha^-1^)	2022-15-03	2022-02-05	2023-18-04	2023-18-04	2023-01-06
Herbicides (pre-sowing)					
Clinic pro (5.0 L ha^-1^; glyphosate)	–	2022-15-03	–	–	–
Herbicides (pre-emergence)					
Proman (1.5 L ha^-1^; metobromuron)	2022-05-05	2022-05-05	2023-25-05	2023-25-05	2023-01-06
Centium (0.2 L ha^-1^; clomazon)	2022-05-05	2022-05-05	2023-25-05	2023-25-05	2023-01-06
Frontier Elite (1 L ha^-1^; dimethenamide)			2023-25-05	2023-25-05	2023-01-06
Arundo (1 L ha^-1^; dimethenamide)	2022-05-05	2022-05-05	–	–	–
Herbicides (post-emergence)					
Corum (1.25 L ha^-1^; bentazon and imazamox)	–	–	2023-21-06	2023-21-06	–
Dash (0.625 L ha^-1^; C-65 methyl esters and klearfac AA-270)	–	–	2023-21-06	2023-21-06	–
Insecticides					
Split (0.42 L ha^-1^; deltamethrin)	2022-29-06	2022-29-06	–	–	–
Karate Zeon (0.075 L ha^-1^; lambda-cyhalothrin)	–	–	–	–	2023-01-08
Acaricides					
Floramite 240 SC (0.4 L ha^-1^; bifenazate)	–	–	–	–	2023-01-08
Fungicides					
	–	–	–	–	–

Due to seed unavailability, RGT Shouna was replaced by Acardia in 2023. In addition, a third early maturing soybean variety, Hermes, was added (**Table 1**). Commercial *Bradyrhizobium* strains G49 (Biodoz^®^, De Sangosse) and 532C (HiStick^®^, BASF) served as reference inoculants. All strains were cultivated as described for the pot trials. In 2022, seeds were coated with bacterial strains in the exponential growth phase (OD_600_ 0.01), mixed with Signum^®^ adhesive (Rhizobacter) at a 15:1 ratio (estimated concentration: 10^6^ CFU mL^-1^; García Méndez et al., 2025). In 2023, both liquid coating and a novel vermiculite-based coating were tested; only the latter resulted in successful nodulation and was retained for data analysis. *Bradyrhizobium*-vermiculite mixtures were applied using a Satac ConceptML 2000 coater with carboxymethyl cellulose up to one month before sowing. Coated seeds were stored at 4 °C and acclimated to room temperature within 24 hours before sowing. **Table 2** summarizes the CFU per seed for the vermiculite-coated seeds at the time of coating and sowing.

**Table 2:** Rhizobia concentrations on vermiculite-coated seeds of three soybean varieties (Acardia, Hermes and Lenka).

*Bradyrhizobium* strain	CFU seed^-1^ (D_0_)			CFU seed^-1^ at sowing (2023-25-05)^(1)^			CFU seed^-1^ at sowing (2023-01-06)^(2)^			
	Acardia	Hermes	Lenka	Acardia	Hermes	Lenka	Acardia	Hermes	Lenka
Non-inoculated control	0	0	0	0	0	0	0	0	0
G49	3.76 × 10^6^	4.78 × 10^6^	3.29 × 10^7^	9.48 × 10^5^	3.23 × 10^5^	9.04 × 10^5^	2.95 × 10^4^	1.90 × 10^5^	9.13 × 10^4^
532C	7.76 × 10^6^	6.04 × 10^6^	1.92 × 10^7^	2.37 × 10^6^	4.97 × 10^5^	1.95 × 10^5^	9.07 × 10^4^	6.85 × 10^4^	7.11 × 10^4^
521_C7_N1.3	1.89 × 10^6^	1.46 × 10^6^	1.82 × 10^7^	6.32 × 10^5^	5.33 × 10^5^	2.82 × 10^6^	3.39 × 10^4^	4.25 × 10^4^	6.20 × 10^4^
590_E5_N4.2	2.24 × 10^6^	5.29 × 10^5^	2.19 × 10^7^	1.58 × 10^5^	1.47 × 10^5^	1.88 × 10^5^	3.66 × 10^4^	1.42 × 10^4^	1.02 × 10^4^
1200_B8_N1.2	1.78 × 10^5^	8.24 × 10^5^	1.15 × 10^7^	1.35 × 10^5^	6.10 × 10^4^	3.23 × 10^4^	1.16 × 10^4^	3.05 × 10^4^	1.03 × 10^4^

Data represents the mean CFU count at the time of coating (D_0_) and on the day of sowing for the different *Bradyrhizobium* strains. Vermiculite coated seeds were stored at 4 °C until 24 hours before sowing.

^(1)^sowing date of Merelbeke_2023 and Melle_2023

^(2)^sowing date of Poperinge_2023

#### 2.2.2 Field measurements

At R5, nodulation was assessed on ten plants per plot in the gross plot area by counting and drying nodules, following Pannecoucque et al. (2018a). Chlorophyll content was measured at the R5 and R6 stages on the youngest fully developed leaves using the CCM-200 (Opti-Sciences). For both parameters, mean values of ten plants per plot were used for further analysis. At R8, the net plot area (8.75 m²) was harvested with a field trial combine (Wintersteiger Nurserymaster Elite) to determine grain yield, protein content and thousand-kernel weight. Subsamples (~1 kg) were cleaned, if necessary, dried (70 °C, 72 hours) and grain yield adjusted to 15% moisture. Thousand-kernel weight was determined as the mean of four 100-grain subsamples.

#### 2.2.3 High-throughput field phenotyping

**Figure 1:**
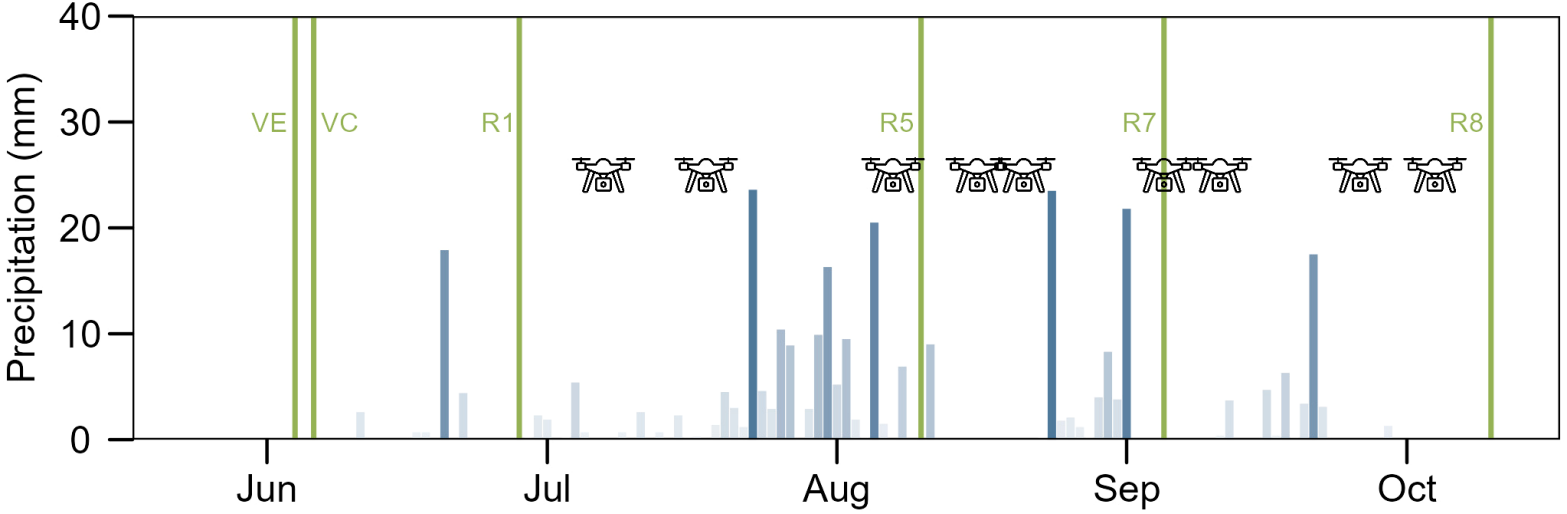
Overview of precipitation (blue bars) and UAV flight data (drone icons) for Merelbeke_2023. High precipitation, wind and cloud cover occasionally prevented weekly flights. Soybean phenological stages are indicated by green vertical lines. Nine UAV flights were performed between growth stages R1 and R8.

**Figure 2:**
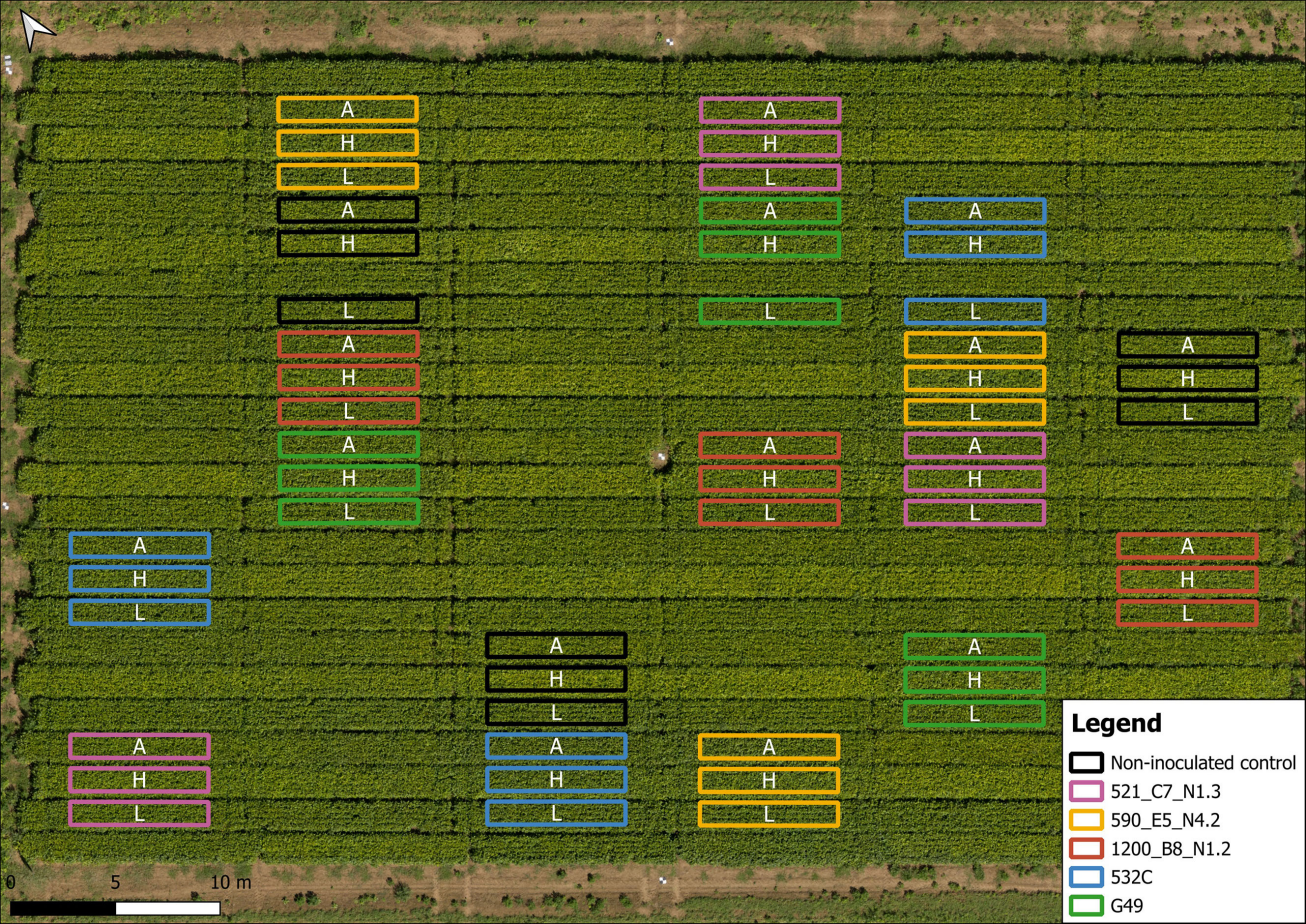
Orthomosaic RGB image of Merelbeke_2023 on 2023-07-08. Plots are annotated with unique colors corresponding with the respective *Bradyrhizobium* strain, while letters indicate soybean varieties: A - Acardia, H - Hermes and L - Lenka. Plots without annotations correspond to treatments inoculated with liquid strains, where no successful inoculation was observed.

The field trial in Merelbeke_2023 was monitored by HTFP using nine drone flights during R1-R8 stages (**Figure 1**). The drone (Matrice 600 Pro, DJI, Shenzhen, China) was equipped with a multispectral sensor (MicaSense RedEdge-MS Dual Camera System, MicaSense, Seattle, USA; 2 cm ground sampling distance (GSD)), and an RGB camera (Sony α6000, 35 mm lens, Sony Corporation, Tokyo, Japan; 0.44 cm GSD). Plot polygons were defined in QGIS v3.40 and processed via a Python-based pipeline (**Figure 2**). Vegetation indices (NDRE, CIG, EVI, SAVI) and canopy height (CH, 90^th^ percentile) were extracted (**Table 3**); final stage (R8) data were excluded due to leaf senescence. Further methodological details follow Borra-Serrano et al. (2020) and Pranga et al. (2021).

**Table 3:** Overview of vegetation indices derived from close-range remote sensing to monitor soybean canopy dynamics throughout the growing season.

Name	Abbreviation	Sensor type	Description
Canopy height [cm]	CH	RGB	Height of the canopy relative to the naked soil (90^th^ quantile)
Normalized difference red edge [-]	NDRE	MS	Chlorophyll content, foliar nitrogen throughout the growing season
Enhanced vegetation index [-]	EVI	MS	Vegetation greenness, reduces atmospheric and soil background effects, especially in dense vegetation
Soil adjusted vegetation index [-]	SAVI	MS	Vegetation cover and biomass in areas with low vegetation density, adjusts for soil brightness effects
Chlorophyll index green [-]	CIG	MS	Chlorophyll content

The UAV was equipped with RGB and multispectral (MS) sensors for all flights.

#### 2.2.4 Chemical analyses

Soil samples at three depths (0–30, 30–60, 60–90 cm) were collected 2–3 months before sowing for organic carbon, mineral nitrogen and pH analysis following ISO standards as previously described by Pannecoucque et al. (2018a) (**Table 1**).

Grain protein content was estimated by NIRS (FOSS XDS) using calibrations based on national datasets containing 5614 samples from 2015-2023. Similar methodology for calibration was used as described by Pannecoucque et al. (2018a), except that in this study NIRS was performed on total soybeans. The standard error of calibration was 8.3 g kg^-1^ for crude protein and the R² of the simple linear regression between reference values and NIRS predicted values of the calibration set was 0.96. The protein estimation by NIRS was validated with Kjeldahl.

### 2.3 Statistical analysis

All data analyses were performed in R version 4.3.2 using the packages lme*4*, *emmeans*, *ggplot2*, *dplyr* and *factoextra* for mixed-model fitting and data visualization. The base models for growth chamber (*Y_GC_*) and field experiments (*Y_F_*) were:


$$Y_{GC}=S+B+S\times B\;|GC\times block$$

$$Y_{F}=S+B+S\times B\;|block$$

where *Y* represents the response variable, and soybean variety (S) and *Bradyrhizobium* strain (B) are fixed effects. The random factor *block* accounted for spatial components within experiments, nested within *GC* for growth chamber experiments. Fixed and random effects are separated by a vertical bar. Mixed-effects models were used to estimate fixed effects, and significance was evaluated by ANOVA. Model assumptions were verified through a multi-step diagnostic process examining Pearson residual and scale-location plots. Data points showing significant deviation from the expected normal distribution (quantile-quantile-plots) or exhibiting excessive leverage on the fitted values were further analyzed. Six observations were found to be outside the plausible range and were removed from the dataset. The regression diagnostics satisfied the requirements of homoscedasticity and normality for the mixed-effects model much better. *Post-hoc* comparisons were performed using Tukey’s HSD test on estimated marginal means (emmeans). Nodulation data of non-inoculated treatments were excluded due to zero variance. Because of differences in inoculation methods and environmental conditions, each field trial was analyzed independently.

## 3 Results

Nodulation, plant and yield traits measured in both growth chamber and field experiments were significantly affected by soybean variety and *Bradyrhizobium* strain. However, interaction effects between these factors were largely absent for most variables (**Table 4**). Therefore, results and figures are presented with primary focus on rhizobial strain performance.

**Table 4:** *P*-values from ANOVA for the effects of soybean variety, *Bradyrhizobium* strain and their interaction on nodulation, plant growth and (post-) harvest traits in growth chamber and field experiments conducted in 2022 and 2023.

Source	Nodule number	Nodule dry weight (g)	Foliar chlorophyll content (CCI)	Shoot dry weight (g)	Grain yield (kg ha^-1^)	Protein content (%)	Protein yield (kg ha^-1^)	Thousand-kernel weight (g)
Growth chamber experiments								
Soybean variety (S)	**0.003**	**< 0.001**	**< 0.001**	**< 0.001**	–	–	–	–
*Bradyrhizobium* strain (B)	**< 0.001**	**< 0.001**	**< 0.001**	**< 0.001**	–	–	–	–
S × B	0.711	0.546	0.438	0.402	–	–	–	–
Field trials								
2022_Bottelare								
S	0.800	0.423	0.062	–	**0.005**	0.480	**0.016**	**< 0.001**
B	**0.003**	**0.002**	**0.014**	–	0.725	0.058	0.269	**0.033**
S × B	0.898	0.625	0.333	–	0.597	0.550	0.409	0.531
2022_Merelbeke								
S	**0.006**	**< 0.001**	**0.031**	–	**0.034**	0.986	**0.026**	**< 0.001**
B	**0.021**	**0.011**	0.078	–	**0.004**	**< 0.001**	**< 0.001**	**0.001**
S × B	0.891	0.138	0.728	–	0.126	0.115	**0.013**	0.177
2023_Melle								
S	0.141	0.053	**< 0.001**	–	**< 0.001**	**< 0.001**	**< 0.001**	**< 0.001**
B	**< 0.001**	**0.017**	**< 0.001**	–	**< 0.001**	**< 0.001**	**< 0.001**	**< 0.001**
S × B	0.774	0.617	0.519	–	0.087	0.883	0.110	0.643
2023_Merelbeke								
S	**0.014**	**< 0.001**	**< 0.001**	–	**< 0.001**	**< 0.001**	**< 0.001**	**< 0.001**
B	**0.002**	**0.021**	**< 0.001**	–	**< 0.001**	**< 0.001**	**< 0.001**	**< 0.001**
S × B	0.181	0.107	0.632	–	**0.002**	0.064	**< 0.001**	0.233
2023_Poperinge								
S	**< 0.001**	**0.041**	**0.038**	–	**< 0.001**	**< 0.001**	0.635	**< 0.001**
B	**0.005**	**0.003**	**< 0.001**	–	**< 0.001**	**< 0.001**	**< 0.001**	**< 0.001**
S × B	0.173	**0.016**	0.806	–	0.850	0.165	0.679	0.490

*P*-values in bold are statistically significant according to Tukey’s HSD (*p* < 0.05).

### 3.1 Effect of indigenous rhizobia on soybean nodulation and early plant vigor in a growth chamber

**Figure 3:**
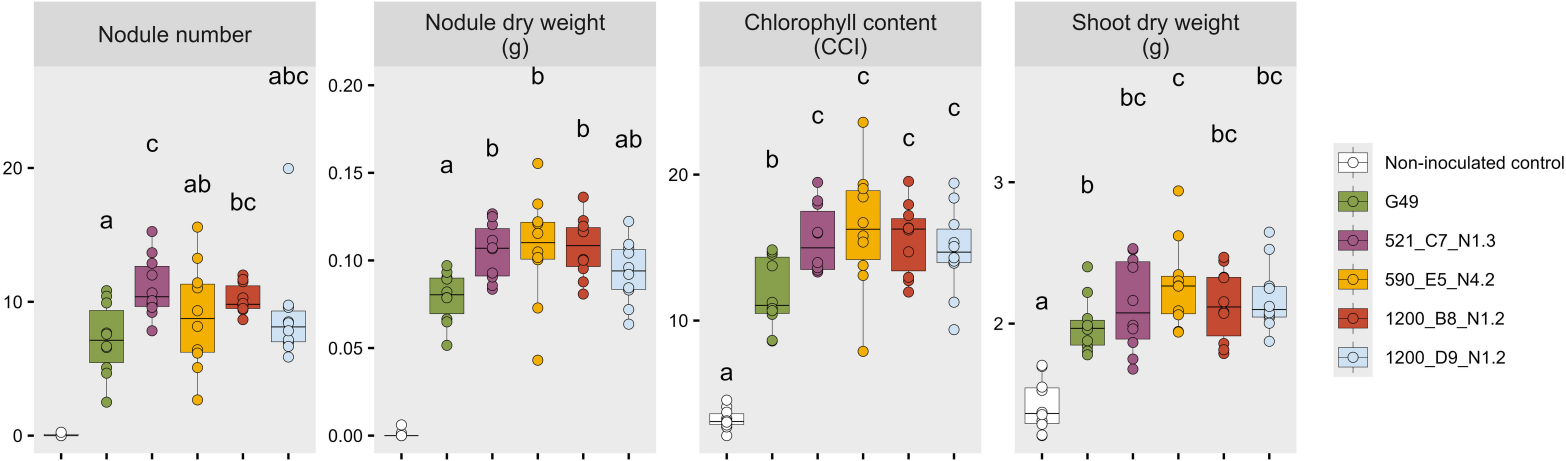
Nodule number, nodule dry weight, foliar chlorophyll content, and shoot dry weight at eight weeks post sowing following inoculation with indigenous (521_C7_N1.3, 590_E5_N4.2, 1200_B8_N1.2 and 1200_D9_N1.2) and commercial (G49) *Bradyrhizobium* strains in two growth chamber experiments. Boxplots annotated with the same letter are not significantly different according to Tukey’s HSD test (*p* < 0.05). The non-inoculated treatment was excluded from statistical analysis of nodule number and nodule dry weight.

All tested indigenous strains (521_C7_N1.3, 590_E5_N4.2, 1200_B8_N1.2, 1200_D9_N1.2) successfully initiated nodulation across soybean varieties, producing healthy, red nodules indicative of active nitrogen fixation. Negative controls showed negligible, nonfunctional nodules. Indigenous strains matched or exceeded the nodulation potential of the commercial strain G49. Notably, 521_C7_N1.3 and 1200_B8_N1.2 induced higher nodule numbers (10.8 and 9.9 vs. 6.7 for G49), while 521_C7_N1.3, 590_E5_N4.2, and 1200_B8_N1.2 achieved higher nodule dry weights (0.105 g, 0.106 g and 0.107 g per plant, respectively vs. 0.080 g for G49) (**Figure 3**; **Supplementary Table 1**).

Six weeks after sowing, inoculated plants developed dark green foliage, indicating improved nutrient status and active nitrogen fixation. In contrast, non-inoculated controls showed nitrogen deficiency symptoms, characterized by pale green-yellow foliage and reduced growth. Inoculation significantly increased chlorophyll content at week eight after sowing compared to non-inoculated controls, with all indigenous strains significantly outperforming G49 (average CCI 15.6 vs. 11.8). Strain 590_E5_N4.2 yielded the highest shoot dry weight (2.27 g), significantly surpassing G49 (1.99 g). Other indigenous strains showed responses comparable to G49. Among the indigenous strains no statistical differences were detected, except for nodule number, which was significantly higher for 521_C7_N1.3 compared to 590_E5_N4.2.

### 3.2 Field trials

#### 3.2.1 Weather conditions during experimental growing seasons

Overall results differed between 2022 and 2023, with markedly lower agronomic outcomes in 2022, primarily due to adverse weather conditions (**Table 5**). Mean growing season temperatures (recorded from May to September) were similar (17.4 °C in 2022 vs. 17.6 °C in 2023), but precipitation patterns contrasted strongly. In 2022, January-April rainfall was only 58.6% of the long-term average (144.5 mm vs. long-term average of 246.6 mm) and July-August precipitation was exceptionally low (22.2 mm vs. long-term average of 163.4 mm). This drought coincided with above-average temperatures, peaking at 26.9 °C in August. In contrast, 2023 was wetter, with January-April rainfall exceeding the long-term average and unusually high summer precipitation (184.9 mm). Peak mean temperatures also exceeded long-term averages, reaching 19.7 °C in June (growth stages VE-VC) and 18.5 °C in September (growth stages R6-R7).

**Table 5:** Monthly mean temperature (maximum temperature in parentheses) and precipitation during 2022 and 2023, compared to the 1991–2020 long-term averages for Belgium.

Month	2022		2023		Long-term mean (1991-2020)	
	Temperature (°C)	Precipitation (mm)	Temperature (°C)	Precipitation (mm)	Temperature (°C)	Precipitation (mm)
January	4.5 (7.1)	49.5	5.8 (8.1)	94.5	3.7 (6.1)	75.5
February	6.8 (10.3)	60.0	5.8 (9.2)	6.9	4.2 (7.1)	65.1
March	8.0 (13.7)	5.5	7.4 (10.9)	110.1	7.1 (10.9)	59.3
April	9.9 (14.9)	29.5	8.7 (13.1)	64.3	10.4 (15.0)	46.7
May	14.8 (20.3)	45.6	13.4 (18.2)	44.3	13.9 (18.4)	59.7
June	17.4 (22.5)	77.9	19.7 (25.5)	31.7	16.7 (21.2)	70.8
July	18.9 (25.0)	7.5	18.6 (23.2)	90.2	18.7 (23.2)	76.9
August	20.7 (26.9)	14.7	18.0 (22.4)	94.7	18.4 (23.0)	86.5
September	15.4 (20.0)	145.2	18.5 (23.7)	61.0	15.2 (19.5)	65.3
October	14.2 (18.5)	27.6	13.8 (17.6)	114.9	11.3 (14.9)	67.8

Data from the Royal Meteorological Institute of Belgium (KMI).

#### 3.2.2 Nodulation and foliar chlorophyll content

**Figure 4:**
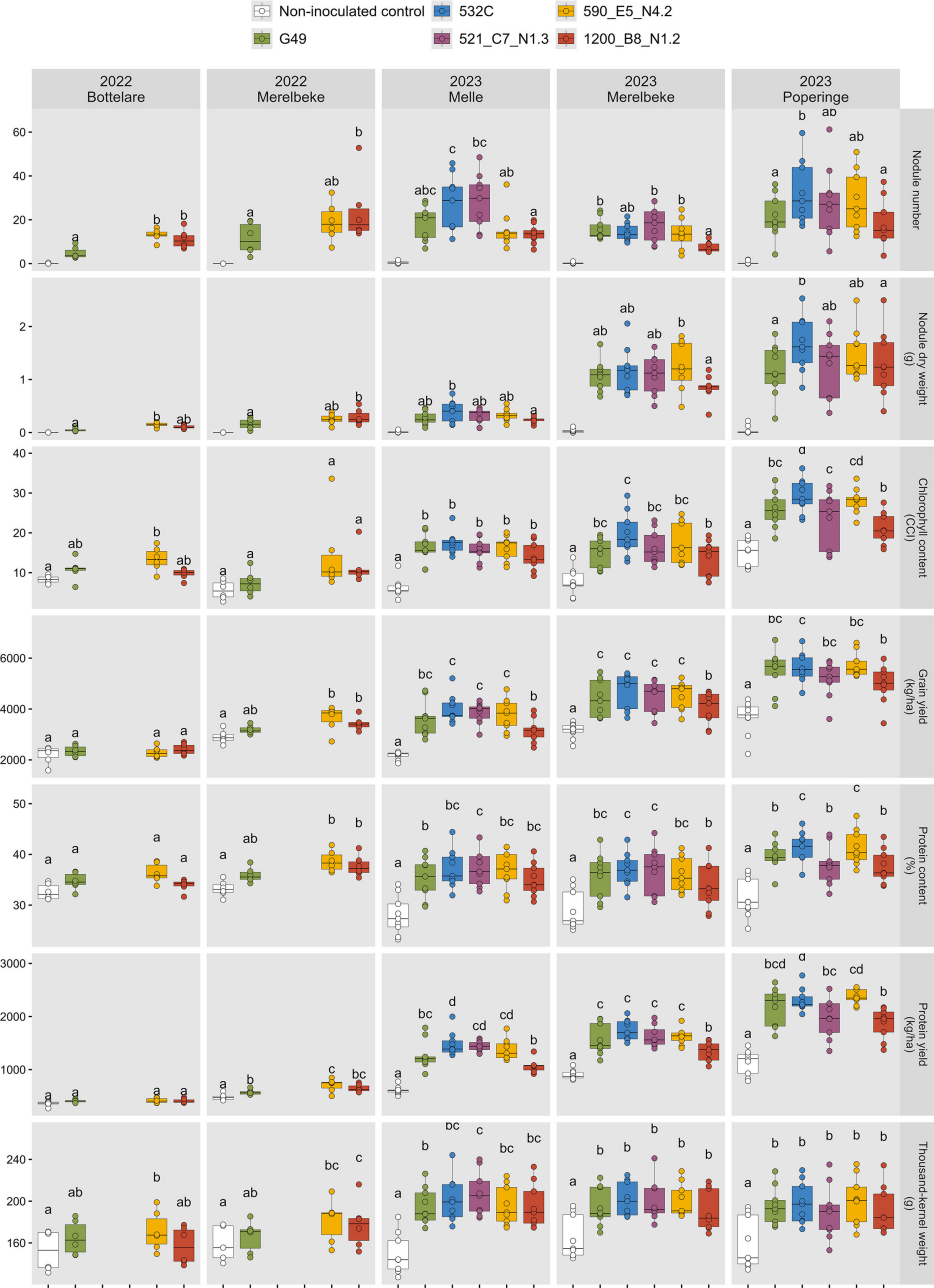
Nodule number, nodule dry weight and chlorophyll content at R5, and yield components (grain yield, protein content, protein yield, and thousand-kernel weight) of soybean following inoculation with indigenous (521_C7_N1.3, 590_E5_N4.2 and 1200_B8_N1.2) and commercial (G49 and 532C) *Bradyrhizobium* strains in the five field trials conducted in 2022 and 2023. Within each location, means annotated by the same letter do not differ significantly according to Tukey’s HSD test (*p* < 0.05). Non-inoculated controls were excluded from statistical analysis of nodulation variables.

Across all five field experiments, inoculation with both commercial (G49, 532C) and indigenous strains (521_C7_N1.3, 590_E5_N4.2, 1200_B8_N1.2) resulted in effective nodulation, with red central tissue in large nodules (**Figure 4**). Only a few small, nonfunctional nodules with white or light-pink central tissue were observed in non-inoculated controls (≤0.4 per plant).

Nodule number and nodule dry weight per plant were significantly affected by strain and variety in Merelbeke_2022, Merelbeke_2023 and Poperinge_2023, while no variety effects were observed in Bottelare_2022 and Melle_2023 (**Table 4**).

In 2022, indigenous strains (590_E5_N4.2, 1200_B8_N1.2) performed similar or even better than G49 for both nodule number and nodule dry weight at the two locations. In 2023, nodule numbers were comparable to 2022, but nodule dry weight per plant was tenfold higher in Merelbeke and Poperinge.

Commercial strain 532C outperformed G49 in Poperinge, while indigenous strains achieved nodulation levels similar to the commercial strains, except 1200_B8_N1.2, which lagged behind 532C in Melle and Poperinge.

In both years, visual differences between inoculated and non-inoculated plots became apparent after R5, with several strains producing darker green foliage than non-inoculated controls. In 2022, G49 did not increase chlorophyll content relative to the non-inoculated controls at either location, whereas indigenous strain 590_E5_N4.2 significantly raised CCI in Bottelare. The same response was observed in Merelbeke_2022 at R3 (data not shown), but the effect was no longer significant at R5 (**Figure 4**).

In 2023, differences were more pronounced. Among the commercial strains, 532C outperformed G49 in Poperinge. Indigenous strains generally matched the commercial strains, except in Poperinge, where 521_C7_N1.3 and 1200_B8_N1.2 did not reach the level of 532C.

#### 3.2.3 Grain yield, protein content and thousand-kernel weight

Grain yield was significantly affected by both variety and strain across most locations, except Bottelare_2022 (**Table 4**). The obtained grain yields for each variety × strain combination is presented in **Supplementary Table 2**. In 2022, yields were lower overall (2316 kg ha^-1^ in Bottelare and 3277 kg ha^-1^ in Merelbeke) (**Supplementary Table 1**) and G49 performed similar to non-inoculated controls (**Figure 4**). Indigenous strains 590_E5_N4.2 and 1200_B8_N1.2 increased yields in Merelbeke. In 2023, yields improved markedly (3460 kg ha^-1^ in Melle, 4233 kg ha^-1^ in Merelbeke and 5143 kg ha^-1^ in Poperinge). Here, 521_C7_N1.3 and 590_E5_N4.2 matched commercial strains, while 1200_B8_N1.2 performed significantly worse than 532C at all sites.

Grain protein content also reflected variety and strain effects in several trials (**Table 4**). In 2022, indigenous strains (590_E5_N4.2 and 1200_B8_N1.2) raised protein levels in Merelbeke (38.5% and 37.7%, respectively vs. 33.2% for non-inoculated plots), while G49 was less effective (36.2%). In 2023, both commercial and indigenous strains raised protein content compared to non-inoculated controls in all trials (37.3% for inoculated vs. 29.2% for non-inoculated plots).

Protein yield, calculated as the product of grain yield and grain protein content, followed similar patterns, more than doubling from 2022 to 2023, and peaking at 1986 kg ha^-1^ in Poperinge. Again, 1200_B8_N1.2 lagged at several sites compared to 523C or the other indigenous strains.

Thousand-kernel weight (TKW) was significantly influenced by variety and strain in all trials, without interaction effects. In 2022, G49 showed no improvement over non-inoculated controls, while 590_E5_N4.2 and 1200_B8_N1.2 significantly increased TKW, with 1200_B8_N1.2 outperforming G49 in Merelbeke. In 2023, TKW increases were more pronounced overall. Indigenous strains performed comparably to commercial strains in Merelbeke and Poperinge, while in Melle, 521_C7_N1.3 exceeded G49. No significant differences were observed between the two commercial strains.

### 3.3 High-throughput field phenotyping as a complementary tool for enhanced field trial analysis

In addition to conventional field measurements, High-Throughput Field Phenotyping (HTFP) was used to extract more detailed temporal insights in Merelbeke_2023. **Figure 2** shows the field layout, illustrating soybean varieties Acardia (A), Hermes (H) and Lenka (L) inoculated with the indigenous *Bradyrhizobium* strains (colored polygons), alongside non-inoculated control plots (black-outlined polygons) on 2023-07-08 (R4). Field plots treated with liquid coatings were excluded from the HTFP analysis.

**Figure 5:**
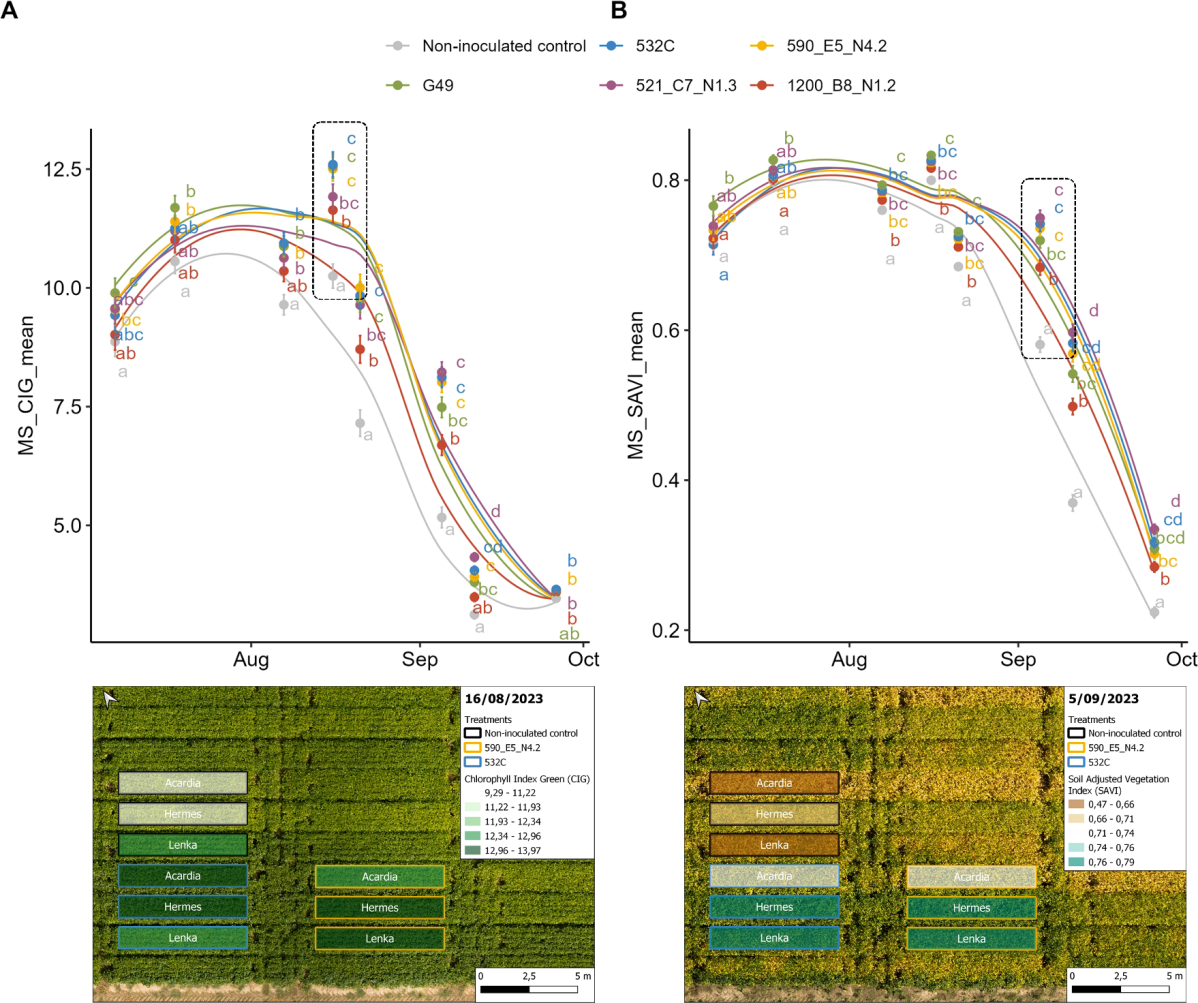
Temporal evolution of UAV-derived vegetation indices throughout the 2023 growing season for the different treatments. ANOVA was performed for each flight to compare treatments. Means annotated by the same letter are not significantly different according to Tukey’s HSD (*p* < 0.05). **(A)** Evolution of chlorophyll index green (CIG) with an RGB image from 2023-16-08. Experimental plots are outlined and semi-transparently filled based on CIG values, displayed a green-scale gradient where higher values indicate greater canopy chlorophyll levels. **(B)** Evolution of soil adjusted vegetation index (SAVI) accompanied with an RGB image from 2023-05-09. Experimental plots are outlined and semi-transparently filled based on SAVI values, shown on a brown-to-green gradient to visualize spatial variation in vegetation cover while minimizing soil background effects.

The temporal dynamics of chlorophyll index green (CIG) and soil adjusted vegetation index (SAVI) were visualized (**Figure 5**). CIG peaked between the second (2023-16-07) and third (2023-07-08) UAV flights, corresponding with pod onset (**Figure 5A**). From mid-August (R5), clear treatment differences appeared. Strains 521_C7_N1.3, 590_E5_N4.2 and 532C maintained significantly higher CIG values during plant senescence, while non-inoculated controls declined rapidly, falling below all inoculated treatments. By mid-September, plants inoculated with strain 521_C7_N1.3 retained the highest plant chlorophyll levels.

SAVI (**Figure 5B**) remained relatively stable from flowering to pod growth (July-August, R1-R5) with differences between inoculation treatments becoming evident at R5 (late August, onset of senescence). Non-inoculated plants showed the earliest and most rapid decline in SAVI. The indigenous strain 1200_B8_N1.2 performed better than non-inoculated controls during senescence but remained consistently below the commercial strains G49 and 532C. Strain 521_C7_N1.3 maintained the highest SAVI values. Overall, rhizobia inoculation delayed senescence and extended the grain-filling period, with SAVI trends closely aligning to CIG dynamics.

At the R5 growth stage, significant differences were found among varieties and strains for all vegetation indices (VI), and significant interaction effects were detected for CIG, NDRE and EVI (**Table 6**). Indigenous strains 521_C7_N1.3 and 590_E5_N4.2 performed similarly to the two commercial strains. Strain 1200_B8_N1.2 showed moderate effects, with higher values for CIG, NDRE, EVI and SAVI compared to non-inoculated plants, although its values remained lower than those observed for the commercial strains. For CH, strain 1200_B8_N1.2 performed the same as the non-inoculated control. These overall patterns correspond well with the performance of the strains as described in previous sections (3.2 Field trials).

**Table 6:** Mean values of canopy height (CH) and chlorophyll-related vegetation indices (VI) - chlorophyll index green (CIG), normalized difference red edge (NDRE), enhanced vegetation index (EVI) and soil adjusted vegetation index (SAVI) - for the different treatments on 2023-21-08.

*Bradyrhizobium* strain	CH	CIG	NDRE	EVI	SAVI	Chlorophyll content (CCI)
Non-inoculated control	0.783 a	7.152 a	0.380 a	0.737 a	0.685 a	7.4 a
G49	0.931 c	9.769 c	0.466 c	0.814 c	0.731 c	15.3 bc
532C	0.861 abc	9.837 c	0.468 c	0.808 c	0.725 bc	19.3 c
521_C7_N1.3	0.892 bc	9.640 bc	0.463 c	0.805 bc	0.725 bc	16.6 bc
590_E5_N4.2	0.886 bc	10.006c	0.471 c	0.803 bc	0.721 bc	17.6 bc
1200_B8_N1.2	0.809 ab	8.706 b	0.432 b	0.781 b	0.711 b	13.8 b
*P*-values							
S	**< 0.001**	**< 0.001**	**< 0.001**	**< 0.001**	**< 0.001**	**< 0.001**
B	**< 0.001**	**< 0.001**	**< 0.001**	**< 0.001**	**< 0.001**	**< 0.001**
S × B	0.920	**0.004**	**0.004**	**0.043**	0.095	0.632
Correlation coefficients with chlorophyll content	**0.54**	**0.80**	**0.80**	**0.78**	**0.75**	1

Means followed by the same letter are not significantly different according to Tukey’s HSD (*p* < 0.05). ANOVA results for soybean variety (S), *Bradyrhizobium* treatment (B) and their interaction effect (S×B) are shown, along with Pearson correlation coefficients between each VI and chlorophyll content (measured on 2023-31-08). Bold values indicate significant differences or correlations (Tukey’s HSD, *p* < 0.05).

Strong positive correlations were observed between manually measured foliar chlorophyll levels at R5 and UAV-derived VI obtained on 2023-21-08 (**Table 6**). The chlorophyll-related indices (CIG, NDRE, EVI and SAVI) showed the strongest correlations with chlorophyll content (r = 0.75-0.80), while CH exhibited a moderate correlation (r = 0.54). All correlations were statistically significant (*p* < 0.05).

## 4 Discussion

Our experiments demonstrated variation in nodulation and yield characteristics between early maturing soybean varieties and indigenous rhizobia strains. Significant variety × strain interaction effects were absent for most trials and parameters, although such an effect was detected in a few instances. The overall pattern indicates that the tested strains were capable of nodulating a range of soybean varieties, reflecting a broad symbiotic adaptability. This contrasts with previous reports of genotype-specific interactions influencing symbiotic effectiveness and plant performance (Omari et al., 2022; Wang et al., 2018; Zimmer et al., 2016). However, the relatively limited set of varieties included in our study, and the fact that only one variety was common for both years, may not fully capture the multifactorial nature of soybean × rhizobia interactions (Agoyi et al., 2017).

Under controlled growth chamber conditions, we confirmed earlier findings (García Méndez et al., 2025), that indigenous Belgian rhizobial strains successfully formed nodules. Indigenous strains performed as well as, or in some cases even better than, the commercial strain G49 in terms of nodulation efficiency. Furthermore, plants inoculated with indigenous strains exhibited significantly higher foliar chlorophyll contents and equal or greater shoot dry weights, underscoring their potential to enhance plant growth under controlled conditions. A direct comparison among the three indigenous strains showed no significant differences, as they performed equally well in growth chamber experiments.

By contrast, field trials revealed considerably more variable results across years and locations, highlighting the strong influence of environmental conditions on strain performance in terms of nodulation and yield characteristics. Differences between years cannot be attributed solely to environmental factors, as inoculation methods also differed between seasons. In 2023, liquid-coated plots performed poorly, with several plots failing to form nodules (data not shown), likely due to issues during rhizobial cultivation or seed coating. As a result, the direct effects of inoculation method cannot be disentangled from climatic influences, and the observed interannual variation reflects a combination of climatic and methodological factors.

The environmental sensitivity of rhizobial performance was particularly evident when comparing the two seasons. The year 2022 was one of the driest since 1991, with rainfall deficits before sowing and during flowering and pod onset. Drought during early growth stages likely impaired rhizobial root colonization and nodule initiation (Kunert et al., 2016; Lumactud et al., 2023), as reflected in the tenfold lower nodule dry weight compared to 2023. Drought during later stages further limited BNF activity and nitrogen fixation (Pitumpe Arachchige et al., 2020; Sinclair et al., 2007), finally reducing yields and protein contents of harvested soybeans (de Freitas et al., 2022). In Bottelare, where precipitation during the growing season was the lowest, yield and protein content did not differ significantly by inoculation. Conversely, in Merelbeke, strains 590_E5_N4.2 and 1200_B8_N1.2 increased grain yield, protein content and thousand-kernel weight relative to non-inoculated controls, whereas G49 failed to do so, suggesting better local adaptation of indigenous strains. This aligns with previous observations that G49 performs poorly under drought stress (Pinochet et al., 1994).

In contrast, 2023 was characterized by abundant rainfall prior to sowing, followed by favorable moisture and temperature conditions during flowering and grain filling. Grain yields in Poperinge reached nearly six tons per hectare, which is well above the average soybean yields of early maturing varieties in Europe (Rotundo et al., 2024). Across both seasons, mean grain yields exceeded those reported for MG000 varieties under rainfed conditions in Northeast Germany (1.22 t ha^-1^; Omari et al., 2022) and were comparable to or higher than yields observed in Central and Southern Germany (3.39 t ha^-1^; Sobko et al., 2020) and France (3.56 t ha^-1^; Lamichhane et al., 2020). These studies highlight substantial interannual and site-specific variability, consistent with the yield fluctuations observed in the present study.

As water availability and temperature were not limiting factors in 2023, the high productivity in Poperinge may be attributed to favorable soil properties, including the highest organic carbon content and a neutral pH (6.5, compared to 5.5 or 6.1 at the other sites). All tested strains successfully nodulated soybean and significantly improved all measured parameters compared to non-inoculated plots. The suboptimal performance of G49 seen in 2022 was not observed in 2023, while 532C confirmed its suitability for temperate regions (Jarecki et al., 2024; Prusiński et al., 2020). Among the indigenous strains, 590_E5_N4.2 and 521_C7_N1.3 performed best across environments, whereas 1200_B8_N1.2 performed well during the dry season of 2022, but showed significantly poorer results under optimal growth conditions in 2023. Additional research is needed to further explore the possible resilience of strain 1200_B8_N1.2 to drought.

Compared to our previous report (García Méndez et al., 2025), the inclusion of additional physiological parameters and a second year of field data allowed a more comprehensive evaluation of strain performance under diverse soil and weather conditions. Notably, the commercial strain G49, and in 2023 also 532C, two widely used rhizobium strains for soybean inoculation in cool environments in respectively France and Canada (Hume and Shelp, 1990; Pinochet et al., 1993), also showed varying performances across years and locations. As none of the experimental fields had a history of soybean cultivation before and no major pest or disease pressure was observed, variation is most likely due to differences in strain adaptation to environmental and soil-related factors (Chalk et al., 2010; Kibido et al., 2020; Peters et al., 2005; Santachiara et al., 2019; Yeremko et al., 2025). In Northwestern Europe, characterized by short and cool growing seasons, temperature and precipitation are key factors for optimal soybean growth (Schmidt et al., 2015; Staniak et al., 2023).

UAV-based HTFP proved to be an effective complementary tool to assess rhizobia inoculation effects. Compared with traditional field measurements, HTFP enabled earlier detection of treatment differences, as variations in vegetation indices were already detected at flowering time, and offered greater temporal resolution by capturing continuous growth dynamics throughout the season. While UAV-derived metrics closely aligned with ground-based measurements, confirming their reliability (Borra-Serrano et al., 2020; Saleem et al., 2022), they currently can be seen as a complementary approach: some key traits, such as nodule number, nodule dry weight and seed yield and composition, still require destructive sampling. Nevertheless, HTFP can reduce the frequency and scale of destructive measurements by identifying critical time points and highlighting treatments of interest. Using this approach, indigenous strains 521_C7_N1.3 and 590_E5_N4.2 were shown to establish comparable canopy and biomass development to commercial strains, demonstrating the method’s sensitivity in detecting strain-specific growth differences.

Overall, while growth chamber experiments indicated consistently strong nodulation and biomass responses of indigenous strains, field trials revealed greater variability, emphasizing the need for multi-environment testing (O’Callaghan et al., 2022). Our findings suggest that indigenous rhizobia may offer greater resilience across diverse weather conditions or soil properties compared to commercial strain G49 which appears more sensitive to environmental stress. Ongoing research aims to expand testing across a wider range of environments and integrate detailed environmental characterizations to disentangle the complex interactions among soybean genotype, rhizobia strain and field conditions.

## 5 Conclusion

In summary, this study demonstrated that indigenous *Bradyrhizobium* strains are equally effective, and in some cases superior, in nodulating early maturing soybean compared to self-cultured strains of the commercial inoculants Biodoz^®^ (strain G49) and HiStick^®^ (strain 532C). The indigenous strains performed well with a subset of early maturing MG000 varieties both in growth chamber and field experiments, although variation in nodulation efficacy and yield potential was evident under field conditions. Further multi-environmental and multi-variety trials are needed to confirm these findings. Nonetheless, results indicate that indigenous rhizobia have strong potential to enhance yield and protein content in local soybean production, representing a critical step towards establishing profitable cultivation in Belgium.

## Data Availability

The raw data supporting the conclusions of this article will be made available by the authors, without undue reservation.
