# Supplementary material for: Indigenous Bradyrhizobium strains enhance nodulation and yield of early maturing soybean in Belgium
Source: Front Plant Sci. 2026 Mar 6;17:1748102. doi: 10.3389/fpls.2026.1748102 (PMC13002570; doi:10.3389/fpls.2026.1748102)
Supplement: Supplementary file 1 [file Table1.docx]

Supplementary Material

Supplementary Table 1 Mean overall results of nodule number, nodule dry weight, chlorophyll content, shoot dry weight, grain yield, protein content, protein yield and thousand-kernel weight following inoculation with indigenous (521_C7_N1.3, 590_E5_N4.2, 1200_B8_N1.2 and 1200_D9_N1.2) and commercial (G49 and 532C) *Bradyrhizobium* strains in growth chamber trials and five field experiments.

|  |  | *Bradyrhizobium* strain | Nodule number | Nodule dry weight (g) | Chlorophyll content (CCI) | Shoot dry weight (g) | Grain yield  (kg ha^-1^) | Protein content (%) | Protein yield (kg ha^-1^) | Thousand-kernel weight (g) |
| --- | --- | --- | --- | --- | --- | --- | --- | --- | --- | --- |
| Growth chamber experiments | | Non-inoculated control | - | - | 3.2 | 1.42 | - | - | - | - |
|  |  | G49 | 6.7 | 0.080 | 11.8 | 1.99 | - | - | - | - |
|  |  | 521_C7_N1.3 | 10.8 | 0.105 | 15.5 | 2.13 | - | - | - | - |
|  |  | 590_E5_N4.2 | 8.0 | 0.106 | 16.3 | 2.27 | - | - | - | - |
|  |  | 1200_B8_N1.2 | 9.9 | 0.107 | 15.6 | 2.13 | - | - | - | - |
|  |  | 1200_D9_N1.2 | 8.5 | 0.094 | 14.8 | 2.19 | - | - | - | - |
| 2022 | Merelbeke | Non-inoculated control | - | - | 5.1 | - | 2854 | 33.2 | 469 | 156 |
|  |  | G49 | 10.9 | 0.178 | 7.5 | - | 3254 | 36.2 | 591 | 167 |
|  |  | 590_E5_N4.2 | 16.3 | 0.222 | 14.8 | - | 3611 | 38.5 | 701 | 178 |
|  |  | 1200_B8_N1.2 | 24.6 | 0.317 | 9,9 | - | 3390 | 37.7 | 635 | 179 |
|  | Bottelare | Non-inoculated control | - | - | 8.3 | - | 2208 | 32.6 | 358 | 153 |
|  |  | G49 | 4.6 | 0.046 | 10.8 | - | 2346 | 34.7 | 406 | 165 |
|  |  | 590_E5_N4.2 | 12.9 | 0.146 | 13.4 | - | 2298 | 36.3 | 417 | 171 |
|  |  | 1200_B8_N1.2 | 10.8 | 0.105 | 9.7 | - | 2411 | 33.9 | 409 | 157 |
| 2023 | Melle | Non-inoculated control | - | - | 6.8 | - | 2209 | 28.3 | 634 | 150 |
|  |  | G49 | 18.7 | 0.290 | 15.7 | - | 3662 | 35.0 | 1282 | 193 |
|  |  | 532C | 26.5 | 0.390 | 18.0 | - | 4075 | 37.1 | 1505 | 202 |
|  |  | 521_C7_N1.3 | 28.3 | 0.337 | 15.6 | - | 3808 | 37.6 | 1418 | 208 |
|  |  | 590_E5_N4.2 | 14.9 | 0.313 | 16.8 | - | 3832 | 36.8 | 1397 | 196 |
|  |  | 1200_B8_N1.2 | 11.4 | 0.208 | 14.6 | - | 3176 | 35.4 | 1122 | 197 |
|  | Merelbeke | Non-inoculated control | - | - | 7.4 | - | 3138 | 28.7 | 883 | 165 |
|  |  | G49 | 14.6 | 1.034 | 15.3 | - | 4405 | 35.8 | 1577 | 194 |
|  |  | 532C | 13.7 | 1.201 | 19.3 | - | 4733 | 36.8 | 1722 | 201 |
|  |  | 521_C7_N1.3 | 17.9 | 1.078 | 16.6 | - | 4556 | 36.9 | 1673 | 200 |
|  |  | 590_E5_N4.2 | 14.3 | 1.324 | 17.6 | - | 4537 | 35.7 | 1603 | 200 |
|  |  | 1200_B8_N1.2 | 6.7 | 0.822 | 13.8 | - | 4028 | 33.8 | 1344 | 191 |
|  | Poperinge | Non-inoculated control | - | - | 13.2 | - | 3521 | 30.6 | 1054 | 157 |
|  |  | G49 | 14.3 | 0.851 | 23.3 | - | 5367 | 38.9 | 2091 | 194 |
|  |  | 532C | 37.5 | 1.932 | 32.4 | - | 5872 | 42.1 | 2457 | 201 |
|  |  | 521_C7_N1.3 | 31.9 | 1.541 | 26.7 | - | 5432 | 38.7 | 2111 | 191 |
|  |  | 590_E5_N4.2 | 24.7 | 1.542 | 28.7 | - | 5819 | 41.9 | 2417 | 201 |
|  |  | 1200_B8_N1.2 | 16.7 | 1.124 | 19.4 | - | 4846 | 37.0 | 1784 | 187 |

Supplementary Table 2 Mean grain yields of various early maturing soybean varieties inoculated with self-cultured indigenous (521_C7_N1.3, 590_E5_N4.2, 1200_B8_N1.2 and 1200_D9_N1.2) and commercial (G49 and 532C) *Bradyrhizobium* strains, across five locations in Flanders, Belgium.

|  |  | | *Bradyrhizobium* strain | Grain yield (kg ha^-1^) | | | |
| --- | --- | --- | --- | --- | --- | --- | --- |
|  |  |  |  | RGT Shouna^(1)^ | Lenka | Acardia | Hermes |
| 2022 | | Merelbeke | Non-inoculated control | 2780 | 3022 | - | - |
|  |  |  | G49 | 3241 | 3126 | - | - |
|  |  |  | 590_E5_N4.2 | 3472 | 3798 | - | - |
|  |  |  | 1200_B8_N1.2 | 3355 | 3505 | - | - |
|  |  | Bottelare | Non-inoculated control | 2107 | 2310 | - | - |
|  |  |  | G49 | 2263 | 2429 | - | - |
|  |  |  | 590_E5_N4.2 | 2121 | 2474 | - | - |
|  |  |  | 1200_B8_N1.2 | 2343 | 2480 | - | - |
| 2023 | | Melle | Non-inoculated control | - | 2034 | 2233 | 2261 |
|  |  |  | G49 | - | 3024 | 3491 | 4338 |
|  |  |  | 532C | - | 3546 | 3869 | 4612 |
|  |  |  | 521_C7_N1.3 | - | 3372 | 4043 | 4153 |
|  |  |  | 590_E5_N4.2 | - | 3154 | 3808 | 4337 |
|  |  |  | 1200_B8_N1.2 | - | 2684 | 3129 | 3617 |
|  |  | Merelbeke | Non-inoculated control | - | 2810 | 3337 | 3281 |
|  |  |  | G49 | - | 3647 | 4284 | 5272 |
|  |  |  | 532C | - | 3844 | 5093 | 5281 |
|  |  |  | 521_C7_N1.3 | - | 3743 | 4773 | 5137 |
|  |  |  | 590_E5_N4.2 | - | 3883 | 4753 | 4988 |
|  |  |  | 1200_B8_N1.2 | - | 3301 | 4138 | 4644 |
|  |  | Poperinge | Non-inoculated control | - | 3285 | 4038 | 3634 |
|  |  |  | G49 | - | 4731 | 5992 | 5800 |
|  |  |  | 532C | - | 5085 | 5750 | 5992 |
|  |  |  | 521_C7_N1.3 | - | 4849 | 5581 | 5078 |
|  |  |  | 590_E5_N4.2 | - | 5333 | 6122 | 5823 |
|  |  |  | 1200_B8_N1.2 | - | 4379 | 5123 | 5427 |

*^(1)^Sowing seeds were unavailable in 2023, and the variety was replaced by Acardia*
